# Policy Series: Fifty Plus: Elder Abuse - Past, Present and Future

**DOI:** 10.1093/geroni/igaf122.1478

**Published:** 2025-12-31

**Authors:** Bridget Penhale, Pamela Teaster

## Abstract

This symposium welcomes inquiry and insights between gerontology, geriatrics, law, policy and elder abuse/adult protective services as areas for exploring knowledge and understanding of violence and abuse in later life. In bridging these contributions across violence intervention and prevention, we aim to celebrate 50 years of inquiry in the US on elder abuse – encompassing research, practice, and policy. For this interactive symposium, we have invited contributions to explore previous traditions and inquiry in the field, examine approaches currently being used, and uncover new and future perspectives. Through exploration, discussion, and interaction, we aim to foster collaborations and address challenges and opportunities arising within the field, how these occurred in the past, how they are managed currently, and in the next 10 years - including those that intersect with other areas in gerontology. Laura Mosqueda, M.D. will focus on clinical issues considering several key areas of medical/healthcare practice, including what is known (past work), what is currently happening (present) and what we need to know. From the social work perspective, Georgia Anetzberger, PhD, will explore some of the major developments in this field relating to research and practice, identifying strengths and shortcomings together with potential future initiatives. Robert Blancato, MPA will consider policy issues and the policy framework in elder abuse and elder justice – past, present and future. Finally, Erica Costello, JD will explore legal matters relating to elder abuse, and adoption of criminal and civil responses including examination of perspectives on fatality review teams and restorative justice.

